# Woman Editors-in-Chief of English-Language Medical Journals Published by the Japanese Professional Medical Associations

**DOI:** 10.31662/jmaj.2021-0008

**Published:** 2021-12-15

**Authors:** Kayo Harada, Akihiko Ozaki, Anju Murayama, Aritsune Tsuji, Takashi Miyachi, Kana Yamamoto, Tamae Hamaki, Masamichi Harada, Asaka Higuchi, Ryo Iwamatsu, Yurie Kobashi, Shungo Murai, Kumi Oshima, Hiroaki Saito, Yuki Sonoda, Yuta Tani, Tetsuya Tanimoto

**Affiliations:** 1 Medical Governance Research Institute, Tokyo, Japan; 2 Department of Breast Surgery, Jyoban Hospital of Tokiwa Foundation, Iwaki, Japan; 3 Department of Internal Medicines, Graduate School of Medicine, University of Tokyo, Tokyo, Japan; 4 Navitas Clinic, Tokyo, Japan; 5 Department of Orthopedics, Saku Central Hospital Advanced Care Center, Saku, Japan; 6 Department of Hematology, Jyoban Hospital of Tokiwa Foundation, Iwaki, Japan; 7 Department of Gastroenterology, Sendai Kousei Hospital, Sendai, Japan; 8 Department of Nursing, Jyoban Hospital of Tokiwa Foundation, Iwaki, Japan

**Keywords:** Japan, woman, journal, gender inequality, editors-in-chief

## Introduction

Gender inequality matters in the academic field globally, but Japan still lags behind the global movement; for example, the proportion of women medical doctors in Japan was a mere 21.8% in 2018, the lowest among the 34 countries belonging to the Organization for Economic Co-operation and Development ^(1)^.

To further evaluate women participation and their contribution to the medical endeavor in Japan, we investigated the proportion of women Editors-in-Chief (EICs) in English-language medical journals published by Japanese professional medical associations (PMAs) as an indicator because their decisions on publication directly influence the value and trend of academic fields, especially when published in English for global readers ^(2)^.

## Methods

We identified EICs of English academic journals published by Japanese PMAs registered in the University Hospital Medical Information Network (UMIN), Japan’s largest public research and education network. We subsequently collected data on their name, gender, institution, location, occupation, and specialty from each journal’s website and their officially affiliated websites. Regarding gender, we made a comprehensive judgement based on name conformities and photos listed on the websites and the physician registry website by the Ministry of Health, Labour and Welfare. Also, we focused on the specialty of EICs instead of that of journals. We also confirmed accuracy of the data by contacting journal editorial offices when necessary. All data collection was conducted from April 2019 to August 2020.

Then, we conducted descriptive analyses of the obtained data. All the data analysis was done not on a journal basis, but on an EIC basis. Concretely, we first calculated a proportion of women in each EIC subcategory stratified based on occupation, affiliation, and specialty of EICs. Second, we compared the proportion of women who were medical doctor EICs and non-medical doctor EICs in the entire population and groups stratified with their affiliations. Ethical approval was obtained through the Ethics Committee of the Medical Governance Research Institution (approval number: MG2020-04-20200607).

## Results

Among a total of 1197 UMIN-registered PMAs, 317 (26.5%) published 346 English-language academic journals. We excluded 40 (11.6%) journals; 20 (5.8%) for not being related to medicine, 8 (2.3%) for a double count in the journal collection process, 7 (2.0%) for unknown EICs even after contacting the journal editorial offices, 3 (0.9%) for discontinuance, and 2 (0.6%) for nonexistent EICs. Consequently, we considered 306 (88.4%) journals in the analysis and the 351 individuals who served as EICs in these journals. Among the considered journals, 17 (5.6%) had two EICs, 6 (2.0%) had three, 1 (0.3%) had four, and 1 (0.3%) had 20 EICs. Among all EICs, 6 individuals (1.7%) served as EICs of two journals.

Table 1 shows the characteristics of EICs and the number and proportion of women in each subcategory. Medical doctors accounted for 208 (59.3%), and 268 (76.4%) were university professors. As for specialty, 82 (23.4%) and 69 (19.7%) specialized in basic medicine and internal medicine, respectively. Among the EICs, women accounted for 20 (5.7%), with 6 (30.0%) being medical doctors and 14 (70.0%) being university professors. The most common specialty was psychiatry (3 [15.0%]) and pharmacology (3 [15.0%]). Among 41 specialties, 26 (63.4%) did not have any woman EIC, including several major clinical medical fields such as internal medicine and surgery. There was a significantly smaller proportion of women who were medical doctor EICs compared with non-medical doctor EICs (2.9% vs. 9.6%, p = 0.014; Table 2).

**Table 1.** Characteristics of Journal Editors-in-Chief in English Journals Published by the Japanese Medical Association (N = 351).

Variable	Editor-in-Chief	Woman (N [%])
All	351	20 (5.7)
Occupation		
Medical doctor	208	6 (2.9)
Non-medical doctor	136	13 (9.6)
Unknown	7	1 (14.3)
Affiliation		
University (professor)	268	14 (5.2)
University (other professors)^a^	47	4 (8.5)
University (non-professor)	4	1 (25.0)
Other	32	1 (3.1)
Specialty^b^		
Basic medicine	82	6 (7.3)
Internal medicine	69	0 (0)
Psychiatry	26	3 (11.5)
Surgery	20	0 (0)
Dental medicine and dental surgery	19	1 (5.3)
Pathology	16	0 (0)
Medical engineering	11	0 (0)
Orthopedic surgery	10	1 (10.0)
Radiational oncology	10	1 (10.0)
Veterinary medicine and zoology	10	1 (10.0)
Social medicine	8	1 (12.5)
Neurosurgery	7	0 (0)
Pediatrics	6	0 (0)
Gynecology and obstetrics	5	1 (20.0)
Anesthesiology and emergency medicine	4	0 (0)
Bromatology	4	0 (0)
Dermatology	4	0 (0)
Forensic medicine	4	0 (0)
Ophthalmology	4	0 (0)
Other^c^	32	5 (15.6)

^a^ Other professors include the assistant professor, associate professor, clinical professor, emeritus professor, specially appointed professor, and visiting professor. ^b^ This represents specialties of the Editors-in-Chief considered, instead of fields of medical journals. Consequently, in total, 122 Editors-in-Chief worked for journals with specialties that differ from the fields of journals. ^c^ Other includes rehabilitation, oriental medicine, environmental ecology, nursing, otorhinolaryngology, iPS and regenerative medicine, genetic medicine, plastic surgery, preventive medicine, urology, clinical laboratory, sports medicine, medical safety, medical ethics, palliative care, and health science.

**Table 2.** Characteristics of Journal Editors-in-Chief (N = 344)^a^ with a Classification of Medical Doctors and Non-Medical Doctors.

Variable	Medical Doctor		Non-Medical Doctor		
	Editor-in-Chief	Woman in each subcategory of editor (N [%])	Editor-in-Chief	Woman in each subcategory of editor N (%)	p-value^b^
All	208	6 (2.9)	136	13 (9.6)	0.014
Affiliation					
University (professor)	159	5 (3.1)	105	9 (8.6)	0.089
University (other professors)^c^	26	1 (3.8)	21	3 (14.3)	0.311
University (non-professor)	2	0 (0)	1	0 (0)	NA
Other	21	0 (0)	9	1 (11.1)	0.300

^a^ We excluded seven Editors-in-Chief from unknown occupations. ^b^ Fisher’s exact text. ^c^ Other professors include the assistant professor, associate professor, clinical professor, emeritus professor, specially appointed professor, and visiting professor.

## Discussion

Only 6.2% of English medical journals in Japan had women EICs, which is considerably smaller than 12%-18% of similar studies reported from western countries ^(3), (4)^. Particularly, there was only 2.9% of women among medical doctor EICs.

There are two possible reasons. First, the presence of mentors can be associated with a successful career development, but women are less likely to have same-gender mentors and role models in the academic societies of Japan. This is because the absolute number of woman researchers, especially high-ranked medical doctors, is extremely low among the upper generation ^(5)^. Second, sexual and gender discrimination or harassment have impaired the careers of Japanese women medical doctors and researchers. Particularly, a recent scandal illustrates the problem, where entrance examination scores of women applicants for medical schools were manipulated to be lower than those of men to restrict their entrance ^(6)^.

As a study limitation, we could not elucidate the proportion of women members of each PMA, but it would also likely be very low considering the patriarchal nature of Japanese society. Still, our findings show that the Japanese academic society has a striking gender inequality regardless of specialties. It would not resolve in the foreseeable future without the implementation of a countermeasure. To improve women participation in the academic fields of Japan, creating better conditions is crucial by assigning women to higher positions in accordance with their skills. Further, considering lessons learned from experiences in leading countries in gender equality, policy-level interventions and activities by advocacy groups are both important to mitigate a dismal situation in the country ^(7)^.

## Article Information

### Conflicts of Interest

Dr. Ozaki received personal fees from Medical Network Systems outside the scope of the submitted work; Dr. Tanimoto received personal fees from Medical Network Systems and Bionics Co. Ltd. outside the scope of the submitted work.

### Acknowledgement

The authors thank Ms. Erika Yamashita for the data collection and Professor Andy Crump for the constructive opinion and English editing.

### Author Contributions

K. Harada had full access to all the data in the study and take responsibility for the integrity of the data and the accuracy of the data analysis.

*Concept design*: K. Harada, Ozaki and Tanimoto.

*Acquisition of data*: K. Harada, Ozaki, Murayama, Tsuji, Miyachi, Yamamoto, M. Harada, Higuchi, Iwamatsu, Kobashi, Sonoda, Tani, Tanimoto.

*Analysis of data*: K. Harada and Ozaki.

*Interpretation of data*: All authors.

*Drafting of the manuscript*: K. Harada, Ozaki, Tanimoto, and Professor Crump.

*Critical revision of the manuscript for impact intellectual content*: All authors.

*Obtained funding*: None.

*Administrative, technical or material support*: None.

*Supervision*: Ozaki and Tanimoto.

### Approval by Institutional Review Board (IRB)

MG2020-04-20200607
